# A new gatekeeper to control oligodendrogenesis

**DOI:** 10.1371/journal.pbio.3002691

**Published:** 2024-07-11

**Authors:** Tim Czopka

**Affiliations:** Centre for Clinical Brain Sciences, The University of Edinburgh, Edinburgh, United Kingdom

## Abstract

The diversity of oligodendrocyte precursor cells (OPCs) is not well understood and is actively discussed in the field. A new study in *PLOS Biology* describes a novel marker for an OPC subpopulation that controls oligodendrogenesis and myelination.

## Abstract 2

The diversity of oligodendrocyte precursor cells is not well understood and is actively discussed in the field. A new study in PLOS Biology describes a novel marker for a subpopulation of oligodendrocyte precursor cells that controls oligodendrogenesis and myelination.

The myelination of central nervous system (CNS) axons to facilitate rapid and energy-efficient nerve conduction is a dynamic process that occurs throughout development and adulthood, as a form of CNS plasticity, and during regeneration of the damaged CNS [1]. New myelin is formed through the differentiation of oligodendrocyte precursor cells (OPCs). These represent an abundant population of approximately 5% of all CNS cells lifelong, which are evenly distributed throughout the grey and white matter, thereby tiling the tissue with their elaborate processes [2]. Given their abundance, it is a long-standing and actively discussed question in the field to ask how diverse this cell population is. Why do some OPCs give rise to myelinating oligodendrocytes, while others do not? Are there subpopulations of OPCs with distinct properties and functions? What defines these subpopulations and how can we distinguish them? There are several reasons why we are still only beginning to gain insights into these questions. One of them is that there has been a notorious lack of markers that allow us to discriminate among OPCs, in order to be able to interrogate their function. A new study by Moghimyfiroozabad and colleagues has identified that the Complement C1q Like 1 (C1ql1) gene is a marker for a subpopulation of OPCs that is crucial for the generation of myelinating oligodendrocytes [3]. Their findings provide insights into the diversity of this cell type and how their lineage progression may be governed.

Moghimyfiroozabad and colleagues report that approximately half of the entire OPC population expresses C1ql1. Interestingly, analysis of single-cell RNA sequencing data revealed that C1ql1-expressing cells can be found across all OPC and pre-myelinating oligodendrocyte clusters and that its expression does not overlap with established markers that define oligodendrocyte lineage cells at different stages of differentiation. Thus, the authors have identified a genuine OPC marker that is only expressed by a subset of cells. In order to address the function of this OPC subpopulation, the authors ablated C1ql1-expressing OPCs using a Cre-Lox approach where Diphtheria Toxin is expressed from the OPC stage onwards, with 2 striking results. First, the authors report a sustained reduction in OPC numbers. This result is unexpected because it is a common feature of OPCs to homeostatically maintain their numbers and to compensate the loss of dying or differentiating OPCs through proliferation of neighbouring cells [4,5]. Second, even though approximately half of the overall OPC population (the C1ql1-negative one) remained intact by the third postnatal week, their model led to a profound and lasting lack of myelination (Fig 1).

**Fig 1:**
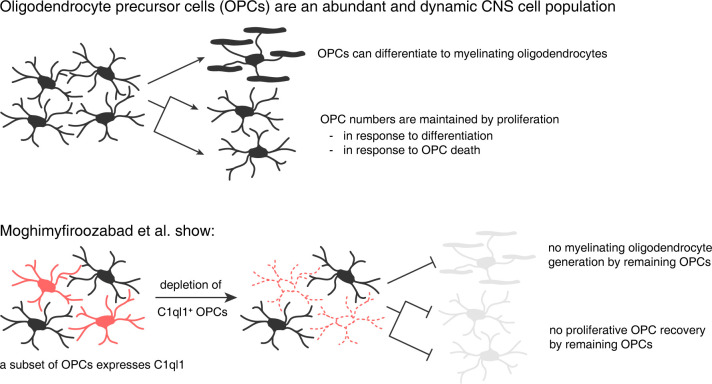
C1ql1 defines a subset of OPCs that is crucial for the generation of myelinating oligodendrocytes. Top: OPCs are abundantly found throughout the CNS where they can differentiate to myelinating oligodendrocytes. OPC numbers are maintained by homeostatic proliferation to replenish cells lost by death or differentiation. Bottom: The authors of the present paper show that C1ql1 labels a subpopulation of OPCs. Ablation of C1ql1-expressing OPCs abolishes myelination and reduces OPC numbers. C1ql1-negative OPCs do not compensate for this loss. C1ql1, Complement C1q Like 1; CNS, central nervous system; OPC, oligodendrocyte precursor cell.

What do these results teach us about OPC diversity and the control of their lineage progression? The finding that C1ql1-negative OPCs neither compensate the reduced OPC numbers nor that they directly give rise to myelinating oligodendrocytes is an intriguing result. Although the authors do not provide experimental data to gain deeper insight, it is worth to speculate about the reasons for these phenomena. It may be that the C1ql1-negative OPCs are an entirely distinct subgroup within the OPC population. However, although this is technically possible, it seems to be an unlikely scenario considering that most, if not all, OPCs proliferate at some point and that all OPCs have the principal capacity to differentiate [2,6]. More consistent with these established OPC properties is the authors’ interpretation that C1ql1 expression defines an OPC state within their population. In such model, each OPC acquires a C1ql1-positive state when contributing to oligodendrogenesis by either cell division or differentiation. Consequently, OPCs that are ready to proliferate and/or to differentiate continuously die in the C1ql1-DTA mouse, leading to a permanent reduction in OPC numbers and block of differentiation. Indeed, it was previously shown using clonal lineage tracing experiments in zebrafish that oligodendrogenesis is controlled by homeostatic OPCs that do not directly contribute to myelination, but which rather indirectly do so through activity-dependent control of proliferation and the generation of daughter OPCs, which can readily proceed to myelination [7]. C1ql1 could define this previously described OPC population that serves as a gatekeeper to control oligodendrogenesis.

Looking forward, lineage tracing of OPCs and C1ql1 expression dynamics will provide answers to these imminent questions on the identity and the detailed role of this cell population. Further along, the function of C1ql1 in OPCs is entirely unclear. C1ql1 has established roles as an organiser of synapse specificity [8]. Intriguingly, OPCs form synaptic contacts with neurons [2]. C1ql1 could regulate the formation of these neuron:OPC synapses and provide a signalling hub for activity integration and the regulation of activity-dependent myelination, but also of non-myelinating functions that OPCs are known to have [6]. These and other questions will be exciting to follow up in the future.

## Abbreviations

C1ql1: Complement C1q Like 1
CNS: central nervous system
OPC: oligodendrocyte precursor cell
